# Development of a Genotyping‐in‐Thousands by Sequencing (GT‐Seq) Panel for Identifying Individuals and Estimating Relatedness Among Alaska Black Bears ( *Ursus americanus* )

**DOI:** 10.1002/ece3.71273

**Published:** 2025-04-11

**Authors:** Eleni L. Petrou, Colette D. Brandt, Timothy J. Spivey, Kristen M. Gruenthal, Cherie M. McKeeman, Sean D. Farley, David Battle, Cory Stantorf, Andrew M. Ramey

**Affiliations:** ^1^ U.S. Geological Survey, Alaska Science Center 4210 University Drive Anchorage Alaska USA; ^2^ U.S. air Force, Joint Base Elmendorf‐Richardson JBER Alaska USA; ^3^ Alaska Department of Fish and Game Anchorage Alaska USA

**Keywords:** amplicon sequencing, GT‐seq, individual identification, parentage, population structure, *Ursus americanus*

## Abstract

The management and conservation of large mammals, such as black bears ( *Ursus americanus* ), have long been informed by genetic estimates of population size and individual dispersal. Amplicon sequencing methods, also known as ‘genotyping‐in‐thousands‐by sequencing’ (GT‐seq), now enable the efficient and cost‐effective genotyping of hundreds of loci and individuals in the same sequencing run. Here, we develop a GT‐seq panel for individual identification and kinship inference in Alaska black bears. Using genomic data from restriction site‐associated DNA sequencing of hunter‐harvested bears from Southcentral Alaska (*n* = 85), we identified 170 microhaplotype and single nucleotide polymorphism (SNP) loci that were highly heterozygous in local populations. To enable sexing of individuals, we also included a previously published sex‐linked locus in the GT‐seq panel. We empirically validated the GT‐seq panel using samples collected at different spatial scales. These samples included tissues (*n* = 82) obtained from bears within a small geographic area in Anchorage, Alaska, which were likely to be relatives as well as the hunter‐harvested samples collected from geographically widespread locations throughout Southcentral Alaska. Empirical validation indicated high genotyping success and genotype reproducibility across replicate subsamples. Computer simulations demonstrated that the GT‐seq panel had ample statistical power for distinguishing distinct individuals and first‐order relatives (parent‐offspring and full‐sibling pairs) from unrelated individuals. As a final proof of concept, the panel was used to identify individual bears and close kin sampled from urban and wild habitats in Anchorage, Alaska. We anticipate that the GT‐seq panel will be a useful genomic resource for the monitoring and management of Alaska black bear populations.

## Graphical abstract

In this paper, we develop and validate a novel amplicon sequencing panel for kinship analysis in Alaska black bears using population genomic data. Our method is compatible with high‐throughput sequencing, yielding faster and more accurate genotypes, reduced costs, and efficient data analysis.

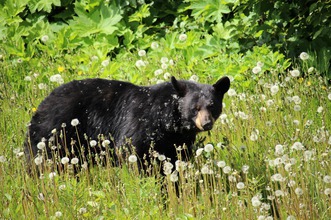

## 1 Introduction

The genetic identification of individuals and their kin has provided numerous insights into the ecology, evolution, and demographic characteristics of wild animal populations. For example, genetic data have been used to describe individual dispersal patterns (Moore et al. 2014), evaluate connectivity between protected areas and harvested populations (Planes et al. 2009; Baetscher et al. 2019), and quantify the reproductive success of dispersing individuals (Peterson et al. 2014). The ability to identify relatives is critical to pedigree reconstruction and the detection of heritable traits (Abadía‐Cardoso et al. 2013), and it has also been used in studies of kin selection and the evolution of cooperative behaviors (Krakauer 2005). Finally, the identification of individuals and relatives is important to natural resource management, as genetic mark‐recapture surveys are frequently used to estimate the size and demographic parameters of harvested populations (Coster et al. 2011; Bravington, Grewe et al. 2016).

In the past, individuals and kin were typically identified using microsatellite markers, which are particularly informative for kinship inference due to their high mutation rate and within‐population variability. The conventional method of genotyping microsatellites is to estimate the size of each allele using capillary electrophoresis. However, this approach is low‐throughput, and technical artifacts such as stutter bands and null alleles can contribute to genotyping errors (reviewed in Guichoux et al. 2011). In addition, integrating data generated by distinct laboratories is challenging, as differences in genotyping equipment can cause slight variations in allele size estimates and genotype calls (Moran et al. 2006). While many of these technical issues have been resolved through the direct sequencing of microsatellites using high‐throughput sequencing (De Barba et al. 2017), distinguishing real alleles from stutter alleles (i.e., genotyping errors) remains challenging in some cases (Zhan et al. 2017; Liu et al. 2024).

More recently, targeted sequencing approaches, commonly known as amplicon sequencing or “genotyping‐in‐thousands by sequencing” (GT‐seq; Campbell et al. 2015), have enabled kinship inference through high‐throughput genotyping of custom panels of single nucleotide polymorphisms (SNPs; Baetscher et al. 2019; May et al. 2020). This method uses highly multiplexed amplification to simultaneously produce genotypes for hundreds or thousands of individuals and loci in a single sequencing run. Because genotyping SNPs from high‐throughput sequence data is relatively straightforward, GT‐seq can achieve high genotyping accuracy at a low cost per sample (Campbell et al. 2015). Although greater numbers of biallelic SNPs relative to microsatellites are required to achieve similar statistical power for relationship inference, SNPs are abundant in the genome. Furthermore, multiple SNPs within a sequence read can be genotyped as a single multiallelic microhaplotype to increase statistical power for kinship identification (Baetscher et al. 2018). Additionally, GT‐seq has been shown to reliably genotype DNA from non‐invasive samples, such as hair or scat (Eriksson et al. 2020; Burgess et al. 2022; Hayward et al. 2022). This capability is particularly significant for studying highly mobile species that are found at low densities and difficult to sample, such as ursids.

**FIGURE 1:**
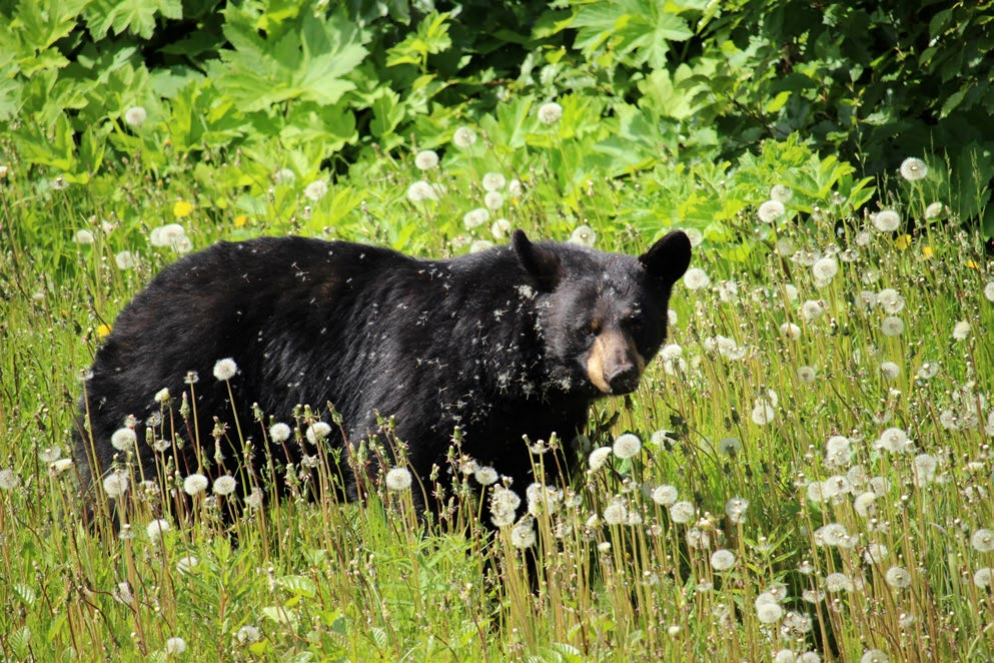
Photograph of an American black bear ( *Ursus americanus* ) in Southcentral Alaska.

Black bears (*Ursus americanus*, Figure 1) are widely distributed across North America and are valued both as a game species and wildlife attraction (Honey et al. 2016). They also serve as an important subsistence resource for some Indigenous communities (Runfola and Naaktgeboren 2020). In many parts of their range, bear populations are recovering from historic overhunting and are impacted by land development and habitat fragmentation (Mattson 1990; Garshelis et al. 2016). Thus, the monitoring of population sizes and demographic rates is important for informing management decisions and regulating harvest.

While black bears typically inhabit forested areas, they are also highly adaptable to human environments and food sources, which increases the likelihood of human‐wildlife interactions or human‐wildlife conflicts (Hristienko and McDonald 2007). As a result, wildlife researchers and managers may seek to identify individual bears for various purposes, including relocation or lethal removal of individuals who repeatedly engage in conflict behaviors or pose threats to public safety. The conservation and management of black bears have long been informed by the genetic identification of individuals using microsatellite markers (Paetkau and Strobeck 1998; Coster et al. 2011; Moore et al. 2014; Reynolds‐Hogland et al. 2022), but currently there are no high‐throughput amplicon sequencing panels available for this species.

In this study, we describe the development of an amplicon sequencing (GT‐seq) panel for Alaska black bears targeting SNP and microhaplotype loci. Using population genomic data, we assess the genetic diversity and population structure in Southcentral Alaska and identify SNPs with high heterozygosity for inclusion in the panel. We assess the panel's statistical power for inferring kinship through simulations and evaluate its performance by genotyping additional independent samples. As a proof of concept, we also present a case study demonstrating the use of the GT‐seq panel to identify individuals and first‐order relatives from samples collected in the municipality of Anchorage, Alaska (‘Anchorage’ henceforth). Our findings show that this GT‐seq panel will enable rapid identification of individuals and close kin in Alaska black bears.

## 2 Methods

### 2.1 Description of Study Area

**FIGURE 2:**
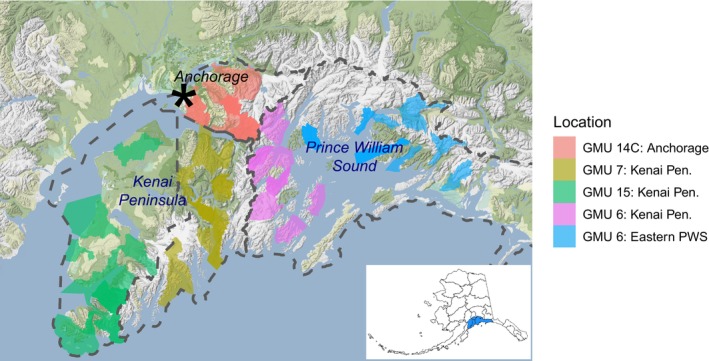
Collection information for hunter‐harvested black bears genotyped with RAD‐seq. The dashed lines in the figure delineate the boundaries of Game Management Units (GMUs), which are further subdivided into Uniform Coding Units (UCUs). The colorful polygons represent 63 distinct UCUs from which bears were harvested. Within GMU 6, UCUs are depicted in two different colors: pink for the Kenai Peninsula and blue for Eastern Prince William Sound. The city of Anchorage is indicated by a star. The small insert map shows the location of sampled GMUs (shown in dark blue) in relation to all other GMUs in Alaska.

Our study area in Southcentral Alaska encompasses approximately 54,203 km^2^ and is composed of four Game Management Units (GMUs; Figure 2) defined by the Alaska Department of Fish and Game. Within this area, black bears inhabit both urban and wild environments, including Anchorage (GMU subunit 14C; 5079 km^2^), the Kenai Peninsula (GMUs 7 and 15; 21,748 km^2^), and the mainland and islands of Prince William Sound (GMU subunits 6C and 6D; 27,376 km^2^). Habitats in this region range from mixed deciduous and coniferous forests to old‐growth forests and estuarine wetlands bordered by muskeg meadows. At higher elevations, the vegetation is predominantly alder (*Alnus* spp.) and willow (*Salix* spp.), eventually transitioning to alpine tundra above the tree line (Viereck and Little 2007). The heavily glaciated Chugach and Kenai Mountains are major landforms in the study area.

### 2.2 Black Bear Sample Collection

Two sets of samples were collected for this study; the first set of samples was used for SNP discovery and primer design, while the second set of samples was used for testing and validation of the GT‐seq panel (Table 1). Samples used for SNP discovery were collected from 96 adult bears harvested by permitted hunters in Southcentral Alaska (Figure 2, Table S1) from 1994 to 2019, and we hereinafter refer to these as “hunter‐harvested” samples. Following the harvest, a pre‐molar tooth was collected from each bear skull and stored until DNA was extracted. Hunter‐harvested samples were collected from several GMUs (Figure 2), including Anchorage (GMU 14C), the Kenai Peninsula (GMU 6, GMU 7, and GMU 15), and Prince William Sound (also GMU 6).

**TABLE 1:** Description of samples used for variant discovery and empirical validation of the GT‐seq panel.

Analysis step	Description of samples	Values
Variant discovery for GT‐seq panel	Number of individuals sequenced	*n* = 96 hunter‐harvested bears
	Number of individuals successfully genotyped with RAD‐seq	*n* = 85 hunter‐harvested bears
Validation of GT‐seq panel	Number of samples sequenced	*n* = 167 (85 hunter‐harvested bears and 82 biopsied bears)
	Number of samples successfully genotyped with GT‐seq	*n* = 160 (78 hunter‐harvested bears and 82 biopsied bears)

Samples used for GT‐seq panel validation were collected from adult and juvenile bears on the Joint Base Elmendorf‐Richardson (JBER) in Anchorage. JBER encircles an area of approximately 260 km^2^, which includes coastal lowlands and forests adjacent to the Chugach Mountains. From 2020 to 2022, biopsy tissue samples (*n* = 82, Table S2) were collected from the hide of free‐ranging bears using a 5 cc Pneu‐Dart biopsy dart (Pneu‐Dart Inc., Williamsport, PA) with a 1.0 cm biopsy needle, fired from a Pneu‐Dart cartridge‐fired gun. All biopsy samples were preserved in Longmire's buffer (Longmire et al. 1997) and stored at 4°C.

### 2.3 RAD‐Seq Library Preparation

We extracted DNA from tooth samples using a NucleoSpin 96 Tissue kit (Macherey‐Nagel, Düren, Germany) protocol modified to accommodate higher volumes of T1 buffer and proteinase K required to process each tooth. To prepare DNA for restriction site‐associated DNA sequencing (RAD‐seq), we followed a slightly modified version of the Ali et al. (2016) protocol which excluded the targeted bait capture step. In brief, 200 ng of DNA from each individual was digested using the restriction enzyme *Sbf*I, and individually barcoded i5 adaptors were ligated to each sample. Following this, an equal volume of each sample was pooled into libraries, which were sheared on a Q500 sonicator (Qsonica, Newtown, CT) for 12 to 14 cycles of 30 s each. Libraries were subsequently purified with Dynabeads M‐280 streptavidin magnetic beads (Invitrogen, Waltham, MA) and AMPure XP beads (Beckman Coulter, Brea, CA) following Ali et al. (2016). DNA was eluted in 55.5 μL of low‐TE buffer and used as input for the NEBNext Ultra DNA Library Prep kit (New England Biolabs, Ipswich, MA), following the manufacturer's instructions and using size‐selection parameters for a 250 bp DNA insert. The final pooled RAD‐seq library was sequenced in conjunction with pooled libraries from three other species on an Illumina NovaSeq 6000 S4 flow cell (2×150 bp) at the University of Oregon Genomics and Cell Characterization Core Facility.

### 2.4 Bioinformatic Analyses for RAD‐Seq Data

Bioinformatic analyses used the computational, storage, and networking infrastructure provided by the Advanced Research Computing Group at the U.S. Geological Survey (Falgout and Gordon 2023). Individual samples were demultiplexed using the *process_radtags* program in *Stacks* v.2.60 (Catchen et al. 2013); low‐quality sequences were removed (using the options *‐‐quality*, *‐‐filter_illumina*, and *‐‐clean*), and sequences were trimmed to a length of 140 base pairs. Subsequently, sequences were aligned to the black bear genome (NCBI RefSeq Assembly: GCF_020975775.1, Srivastava et al. 2019) using the ‐‐very‐sensitive option in *bowtie2* v.2.3.5.1 (Langmead and Salzberg 2012). Successfully paired reads were retained and filtered for mapping quality (*mapq > 20*) with *samtools* v.1.9 (Li et al. 2009), and sequences were realigned around indels using *GATK3* v.3.8 (DePristo et al. 2011).

We genotyped samples and removed PCR duplicates using the *gstacks* program in *Stacks*. To ensure that only high‐quality variants were included in the data, SNPs were filtered for minor allele count (*‐‐min‐mac 3*) and assessed for Hardy–Weinberg equilibrium (HWE) in each sample collection using the *populations* program in *Stacks*. SNPs were retained if they were sequenced in all sample collections (i.e., GMUs) and genotyped in more than 80% of individuals. Additional SNP filtering was done with *VCFtools* v.0.1.16 (Danecek et al. 2011) to remove SNPs with low mean sequencing depth (*−‐min‐meanDP 10*) and retain SNPs with a minor allele frequency (MAF) > 0.05 over all samples. Following Petrou et al. (2019), we screened individuals for intraspecific DNA contamination by calculating individual multilocus heterozygosity (*H*_I_). Samples were removed from the data set if they had *H*_I_ values greater than two standard deviations from the overall mean *H*_I_ calculated using all samples.

To visualize patterns of the population structure among hunter‐harvested samples, we conducted a principal component analysis (PCA) with *adegenet* v. 2.1.8 (Jombart 2008) in R v.4.2.2 (R Core Team 2022). We subsequently explored the number of likely population clusters (*K*) and admixture proportions of individual samples using *Admixture* v.1.3 (Alexander et al. 2009). First, SNPs in linkage disequilibrium (LD) were pruned from the data set (−‐indep‐pairwise 50 10 0.1) using *plink* v.1.90 (Purcell et al. 2007). Using this set of 6630 LD‐pruned SNPs, we conducted a total of 10 replicate runs for *K* ranging from 1 to 5, and we used the cross‐validation (CV) procedure of Alexander and Lange (2011) to evaluate which number of clusters yielded the lowest CV error.

Pairwise population differentiation (*F*_ST_; Weir and Cockerham 1984) between populations was calculated in *StAMPP* v. 1.6.3 (Pembleton et al. 2013), and confidence intervals around *F*_ST_ were estimated using 1000 bootstraps. Finally, we calculated inbreeding coefficients (*F*_IS_) in each population using *hierfstat* v.0.5 (Goudet 2005).

### 2.5 Development of GT‐Seq Panel

Loci with high heterozygosity provide greater statistical power for estimating relatedness (Anderson and Garza 2006). Therefore, we identified SNPs with a global minor allele frequency (MAF) greater than 0.35 and a within‐GMU MAF greater than 0.25, which were genotyped in 95% of individuals. To facilitate primer design, we avoided variants at the ends of sequences and only retained loci with SNPs within 20 base pairs of each other. For SNPs that met these criteria, we retrieved flanking sequences that were 50 base pairs upstream and downstream of each targeted SNP by creating a multi‐way pileup in *samtools* using the black bear genome and all individual sequences. Subsequently, consensus sequences for each amplicon were created with the *consensus* function in *bcftools* v.1.16 (Danecek et al. 2021).

We designed primers for the consensus amplicons with *BatchPrimer3* (You et al. 2008) using the following parameters: target product size of 100 bp (min = 85, max = 140); primer length of 20 bp (min = 18, max = 27); Tm of 60°C (min = 57; max = 63); GC content of 50% (min = 40; max = 60); and no more than four of the same base repeated consecutively (i.e., poly‐X repeats = 4). To minimize off‐target primer interactions or the formation of primer‐dimers, we aligned primer sequences to each other and the black bear genome using *bowtie2* and removed primers that aligned to more than one location on the genome and/or each other. Illumina sequencing primers were subsequently added to the GT‐seq primers following Campbell et al. (2015) with the following modification: the overhang adapter sequence of amplicon‐specific primers (i.e., PCR1 primers) was updated such that individual samples could be labeled with Illumina Unique Dual Index i5 and i7 adapters (Table S3). After these filtering steps, the panel consisted of 172 loci containing 327 SNPs in the RAD‐seq samples (Table S4). Amplicons had 2–8 microhaplotypes per locus (Figure S1) and were distributed across 99 different scaffolds of the black bear genome, with a minimum distance of 30,000 base pairs between loci to reduce the possibility of linkage. To enable sex identification, we also included a sex‐linked locus previously described by Pagès et al. (2009) located on the *SRY* gene on the Y chromosome.

### 2.6 Testing of GT‐Seq Panel

We empirically validated the GT‐seq panel using samples collected at different spatial scales. These samples included hunter‐harvested samples collected from geographically widespread locations throughout Southcentral Alaska (*n* = 85, Table S1), as well as tissues obtained from bears within a small geographic area (JBER), which were more likely to be relatives (*n* = 82, Table S2). DNA was extracted from biopsy samples using the DNeasy Blood and Tissue kit (Qiagen, Germantown, MD) and quantified with a Qubit fluorometer (Thermo Fisher Scientific, Waltham, MA). We normalized samples to a concentration of 20 ng/μL and prepared GT‐seq libraries using the protocol of Campbell et al. (2015). The final fragment size distributions of GT‐seq libraries were quantified with an Agilent ScreenTape device (Agilent Technologies, Santa Clara, CA) using high sensitivity D1000 reagents. To estimate genotyping error rates, we included five replicate subsamples per library, and no‐template controls were used to screen for reagent contamination. Each GT‐seq library contained approximately 90 samples and was sequenced on an Illumina NextSeq 1000 instrument using a P1 flow cell (paired‐end sequencing, 300 cycles), with the addition of 30% PhiX to ensure library complexity.

Rather than genotyping a single high‐heterozygosity SNP on each GT‐seq amplicon, we assembled SNPs on the same sequence read into multiallelic microhaplotypes, as this approach provides increased statistical power for inferring kinship (Baetscher et al. 2018). Microhaplotype genotypes were called at each amplicon following the method of Delomas et al. (2023) that is implemented in the software *microTyper*. As input, this program requires a catalog of known SNP variants as well as individual sequences that have been aligned to GT‐seq amplicons. First, adapters were trimmed from sequencing reads using *trimmomatic* v.0.39 (Bolger et al. 2014), and forward reads were aligned to the GT‐seq amplicons using *bowtie2* (Langmead and Salzberg 2012). Aligned sequences were sorted, indexed, and filtered for mapping quality (‐q 30 and ‐F 4) using *samtools* v.1.19 (Li et al. 2009). Genotype likelihoods were calculated using the *mpileup* command, and SNPs were genotyped using the *call* command and *consensus‐caller* model in *bcftools* v.1.19 (Danecek et al. 2021). We subsequently tested SNPs for deviations from Hardy–Weinberg equilibrium in the hunter‐harvested samples using the exact test based on Monte Carlo permutations of alleles (Guo and Thompson 1992) that is implemented in the R package *pegas* v. 1.3 (Paradis 2010). If a locus was out of HWE in all sample collections, it was filtered from the catalog of variants. Finally, we used *microTyper* to call microhaplotype genotypes for every individual at GT‐seq loci whose sequencing depth was greater than 20 reads. To remove confounded genotypes caused by processes such as index switching or genotyping error, we filtered haplotypes with an allelic read depth ratio of < 0.4, following Baetscher et al. (2019). Individuals with more than 20% missing genotypes were removed from downstream analyses.

To calculate the statistical power of the GT‐seq panel for relationship inference, we estimated the distribution of the log‐likelihood ratio using the importance‐sampling algorithm of Anderson and Garza (2006) implemented in the R program *CKMRsim* (Anderson 2024). First, we simulated individuals of known relatedness (*N* = 10,000 of each category: monozygotic twin/self, parent‐offspring (PO), full‐sibling (FS), half‐sibling (HS), and unrelated (U) pairs) using allele frequencies derived from the microhaplotype genotypes of hunter‐harvested bears. For each pair of simulated individuals, *CKMRsim* then calculated the log‐probability of observing the pair of genotypes conditional on the pair being one of the relationships defined above. These log‐probabilities were subsequently used to estimate distributions of the log‐likelihood ratio for different relationships. For example, to identify possible parent‐offspring pairs, we used the ratio of the probability of a pair's genotypes given that they are parent‐offspring, divided by the probability of the pair's genotypes given that they are unrelated; we use the notation PO/U to describe this log‐likelihood ratio. Following this, the false negative rate (i.e., related individuals misidentified as unrelated) and false positive rate (i.e., unrelated individuals misidentified as relatives) were calculated from the simulated distributions of the log‐likelihood ratios. Simulations and likelihood calculations in *CKMRsim* were made using the true‐genotype‐independent model with an error rate (epsilon) equal to 0.005.

We subsequently used these distributions of the log‐likelihood ratio to identify distinct individuals and first‐order relatives in the empirical samples. First, samples originating from the same individual were identified using the *find_close_matching_genotypes* function, allowing up to ten genotype mismatches per individual. Following this, a hierarchical approach was used in *CKMRsim* to identify parent‐offspring and full‐sibling pairs. Likely parent‐offspring and full‐sibling pairs were identified from unrelated pairs using the PO/U and FS/U log likelihood ratios. Then, fullsiblings were separated from half‐sibling pairs using the FS/HS log‐likelihood ratio, while the PO/FS log‐likelihood ratio was used to differentiate between parent‐offspring and full‐sibling pairs.

Patterns of relatedness were subsequently visualized as networks using the R package *ggraph* v.2.1 (Pedersen 2024). We conducted these analyses separately in the following data sets: (1) biopsy samples genotyped at 170 GT‐seq microhaplotypes; (2) hunter‐harvested samples genotyped at 170 GT‐seq microhaplotypes; and (3) hunter‐harvested samples genotyped with RAD‐seq at 6630 LD‐pruned SNPs. As the same set of hunter‐harvested bears had been genotyped using both RAD‐seq and GT‐seq, we were able to evaluate whether relationship inference depended on the genotyping method and locus set used.

The sex of each individual was determined by counting the number of *SRY* amplicons in each FASTQ sequence file. As this gene is specific to the Y chromosome, males were defined as individuals who had more than 0.01% of total reads aligning to the *SRY* locus. To assess sexing accuracy of the *SRY* locus, we compared results to 30 samples genotyped at more than 50% of GT‐seq loci whose sex had been previously identified using the sex‐linked *zfx/y* gene (Muhlenbruch 2022). Following this, we evaluated whether individual sex was correlated to the number of genetic recaptures using Pearson's chi‐squared test.

## 3 Results

### 3.1 RAD Sequencing for SNP Discovery

A total of 96 individuals collected from four different GMUs in Southcentral Alaska were sequenced using RAD‐seq (Table 2). On average, we obtained 5.5 million sequences per individual (range = 505,087—11,567,565), and there was a 94% overall alignment rate to the black bear genome. After calling genotypes and applying quality filters, we retained 85 individuals genotyped at 56,257 SNPs, whose mean read depth was 13× (range = 10—30×).

**TABLE 2:** Summary information for black bear populations genotyped using RAD‐seq at 56,257 SNPs.

Population	GMUs	*N*_Filt_	*H*_o_	*H*_e_	*F*_IS_	Allelic richness
Anchorage	14 C	30	0.301	0.302	0.007	1.86
Kenai Peninsula	7, 15, 6	43	0.282	0.291	0.033	1.83
Eastern Prince William Sound	6	12	0.253	0.275	0.063	1.75

*Note:* Metadata include information on the population, game management unit (GMU), number of individuals passing genotyping filters (*N*_Filt_), and various population genetic summary statistics (observed heterozygosity, *H*_o_; expected heterozygosity, *H*_e_; inbreeding coefficient *F*_IS_; and allelic richness).

**FIGURE 3:**
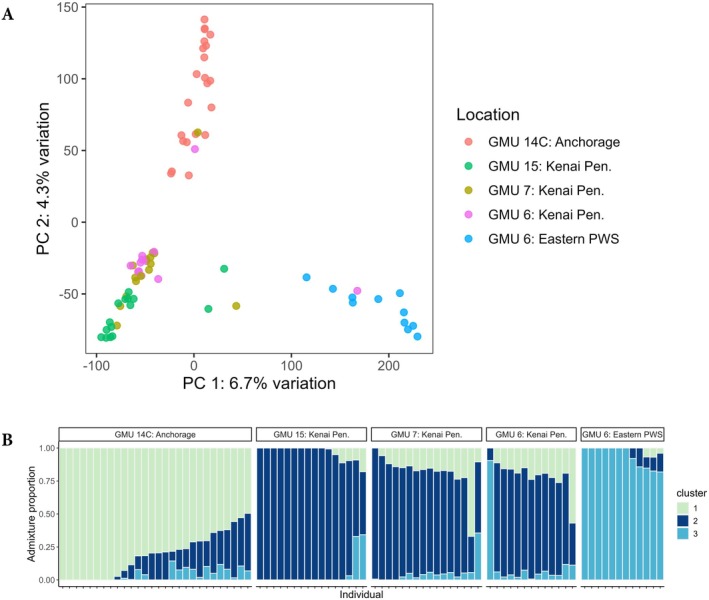
Patterns of population structure for hunter‐harvested black bears genotyped with RAD‐seq. (A) Principal components analysis (PCA) showing individual bear samples as colorful points; colors correspond to sampling location (UCU) as in Figure 2. (B) Admixture proportions estimated for *K* = 3 clusters. Each sample is represented by a bar whose color(s) designates the proportion of ancestry assigned to a particular cluster. Individuals are grouped by the geographic region they were harvested from.

PCA showed that individuals clustered into three major groups which generally corresponded to the geographic areas of sample collection (Figure 3A). The first principal component, explaining 6.7% of the total variation, separated individuals harvested from eastern Prince William Sound (east of College Fjord) from individuals harvested from the Kenai Peninsula and Anchorage. The second principal component, explaining 4.3% of the total variation, separated individuals collected in Anchorage from other samples. Estimates of observed heterozygosity (range = 0.25–0.30) and allelic richness (range = 1.75–1.86) were similar across all populations, and mean per‐locus *F*_IS_ was generally distributed around zero (Figure S2). Pairwise population *F*_ST_ values ranged from 0.038 to 0.120, and all comparisons were statistically significant (*p* < 0.0001; Table 3).

**TABLE 3:** Pairwise population *F*_ST_ calculated for samples genotyped at 56,257 SNPs using RAD‐seq.

Population	Kenai Peninsula	Eastern Prince William Sound
Anchorage	0.038 ***	0.100 ***
Kenai Peninsula		0.120 ***

*Note:* Stars (***) indicate that the value is statistically significant at *p* < 0.0001.

Genetic analyses with *Admixture* indicated that *K* = 3 population clusters provided the lowest CV error (SI Figure 3), and there was support for these groupings across 10 replicate runs. At *K* = 3, individuals were distributed across an Anchorage cluster, a Kenai Peninsula cluster, and an eastern Prince William Sound cluster (Figure 3B), a pattern which is concordant with the PCA results. Bears harvested in GMU 6 were assigned to two different clusters; individuals harvested from the western part of that GMU clustered with individuals from the Kenai Peninsula, while individuals sampled from the eastern part formed the genetically distinct Prince William Sound group. Additionally, we identified many individuals from GMU 14C, GMU 7, and GMU 6 with low levels of admixed ancestry (Figure 3B). These analyses also identified individual dispersal patterns between management units; for example, one male bear whose genetic ancestry was overwhelmingly (90%) from the Prince William Sound population was harvested on the Kenai Peninsula (Figure 3B). We also identified two additional bears (one male and one female) with admixed ancestry predominantly from the Anchorage population (57%–67% ancestry assigned to cluster 1, Figure 3B) who were harvested on the Kenai Peninsula.

### 3.2 Testing of GT‐Seq Panel

**FIGURE 4:**
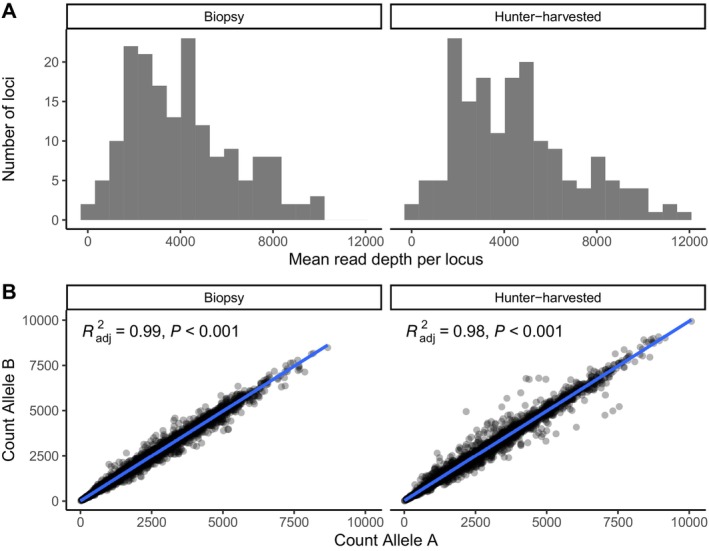
(A) Distribution of mean read depth for GT‐seq loci genotyped in separate sequencing runs conducted using either biopsy or hunter‐harvested black bear samples. (B) Correlation of microhaplotype allelic read depth at heterozygous sites genotyped in biopsy or hunter‐harvested samples.

On average, we obtained 776,274 sequences (range = 292–1,438,412) for each of 167 samples sequenced with GT‐seq, and the overall sequence alignment rate to the black bear genome was 97%. Negative (i.e., no‐template) controls did not produce sequences aligning to the bear genome. After genotypes were called, individuals had 1.8% missing data on average. Six samples had more than 20% missing data and were removed from downstream analyses, as was one locus that failed to amplify across all samples in both sequencing runs. A single locus was out of HWE in every sample collection (*p* < 0.0001) and was removed from the data set. After applying these filters, we retained 160 samples genotyped at 170 autosomal loci and one sex‐linked locus. Average read depth was 4292× per locus (Figure 4A), allelic depth was highly correlated at heterozygous sites (Figure 4B), and mean per‐locus heterozygosity was 0.46.

For the eight individuals sequenced in replicate using GT‐seq, one individual exhibited a single genotype mismatch with its technical replicate, while six individuals matched their replicates perfectly. A single individual did not match its replicate subsample (51% mismatch rate) but was a perfect match for a sample in an adjacent well, indicating that this discordance was the result of aliquoting error during replicate preparation rather than a sequencing error. Females and males that had been previously sexed using *zfx/y* had distinct read count distributions at the *SRY* locus (Figure S4), and all sex identification calls were concordant across both loci.

**FIGURE 5:**
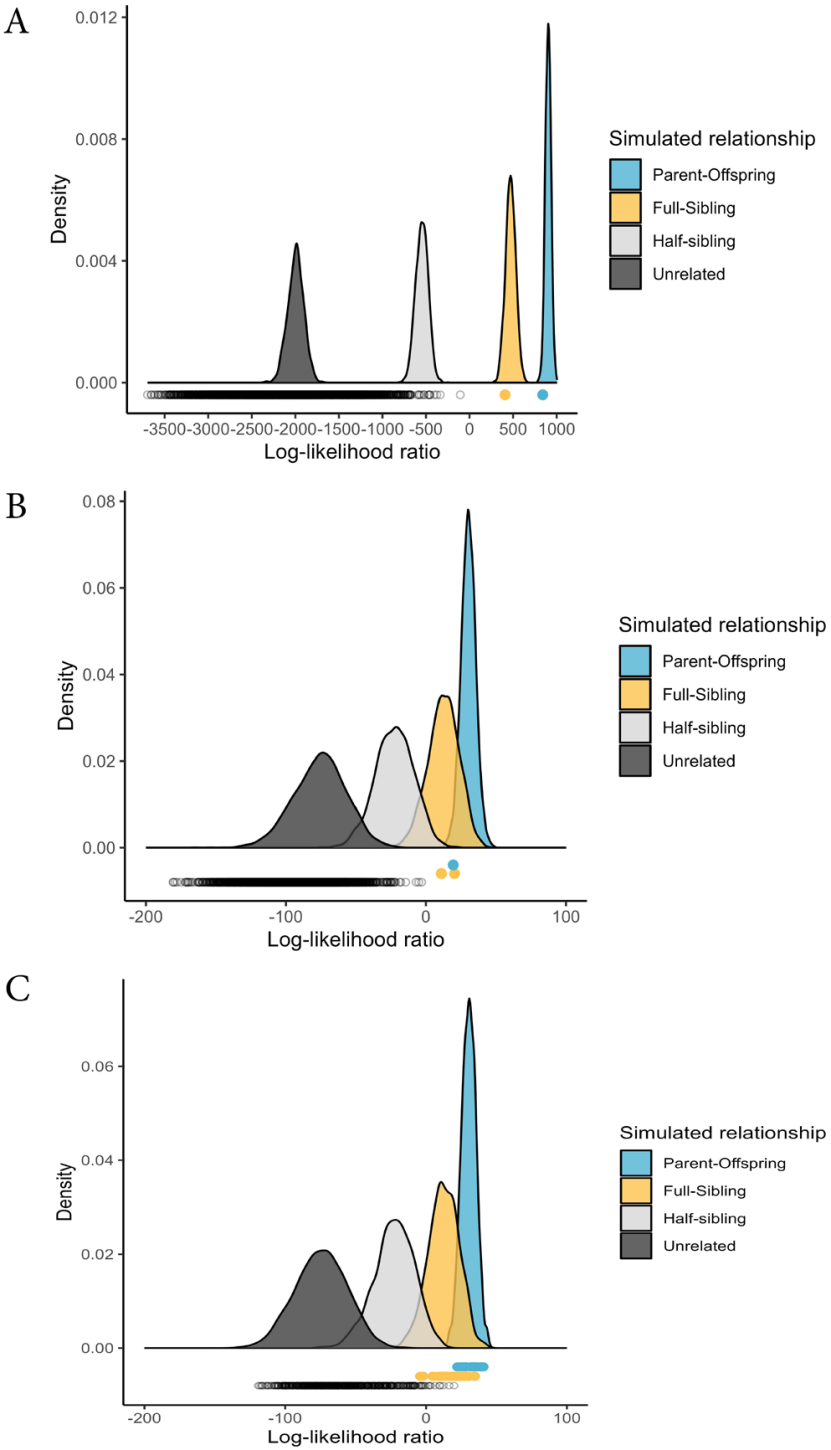
Distribution of the parent‐offspring/unrelated (PO/U) log‐likelihood ratio based on simulated parent‐offspring (PO), full‐sibling (FS), half‐sibling (HS), and unrelated (U) pairs of individuals. Simulations were based on allele frequencies of hunter‐harvested black bears, genotyped at 6630 SNPs using RAD‐seq (A) or 170 microhaplotypes using GT‐seq (B, C). The points below the distributions indicate PO/U ratios calculated for each pair of empirical samples, with points highlighting likely PO (blue), FS (yellow), and all other pairs (black). (A) Hunter‐harvested samples collected from Southcentral Alaska and genotyped with 6630 RAD‐seq SNPs. (B) Hunter‐harvested samples collected from Southcentral Alaska and genotyped with 170 GT‐seq microhaplotypes. (C) Biopsy samples collected from the Joint Base Elmendorf‐Richardson in Anchorage and genotyped with 170 GT‐seq microhaplotypes.

Simulations using microhaplotype allele frequencies indicated that false positive rates for identifying unrelated individuals as monozygotic twins (or replicates of the same individual) and first‐order relatives were very low (Figure S5). At a false negative rate of 0.01, the false positive rate was 1.4×10^−62^ for monozygotic twins, 1.5×10^−10^ for parent‐offspring pairs, and 2.3×10^−7^ for full‐sibling pairs (Figure S5). These simulations were also used to identify specific log‐likelihood ratio thresholds for identifying first‐order relatives. For example, the simulated distribution of log‐likelihood ratios for parent‐offspring pairs ranged from 2 to 49 (Figures 4C and 5B), while the distribution for unrelated pairs ranged from −165 to −12 (Figure 5B,C). Accordingly, parent‐offspring pairs were identified as individuals with a PO/U log‐likelihood ratio > 18 (Figure S6), which corresponds to a false negative rate = 0.01. However, there was significant overlap between the log‐likelihood ratio distributions of parent‐offspring and full‐sibling pairs when only the PO/U log‐likelihood ratio was considered (Figure 5B,C). To differentiate between parent‐offspring and full siblings, we examined the PO/FS log‐likelihood ratio, where there was minimal overlap between the distributions of these relationships (Figure S7). Using a threshold of PO/FS > 3 (represented by red dashed line in Figure S7), we estimated that the probability of misidentifying a true full‐sibling pair as a parent‐offspring pair to be 0.002. This same approach was applied to differentiate between full‐sibling and half‐sibling pairs (Figure S8) as well as full‐sibling and unrelated pairs (Figure S9). Specific log‐likelihood thresholds and false positive rates for all of these pairwise comparisons are reported in Table S5. When hunter‐harvested bears were analyzed using microhaplotypes, we identified three possible first‐order relatives: a parent‐offspring and full‐sibling pair from Anchorage as well as a pair of full siblings from Prince William Sound (Table S6). Repeating these analyses using 6630 LD‐pruned SNPs genotyped with RAD‐seq identified the same parent‐offspring and full‐sibling pairs from Anchorage (Figure 5A), but the pair of individuals from Prince William Sound was identified as likely second‐degree relatives instead. As expected with hunter‐harvested bears, there were no samples with matching genotypes, indicating that all samples originated from distinct individuals. There were similar numbers of females and males (*n*_female_ = 42, *n*_male_ = 37) harvested, based on the *SRY* locus. However, there were eight instances of disagreement between *SRY* sex and hunter‐reported sex, which were distributed evenly between the two sexes.

**FIGURE 6:**
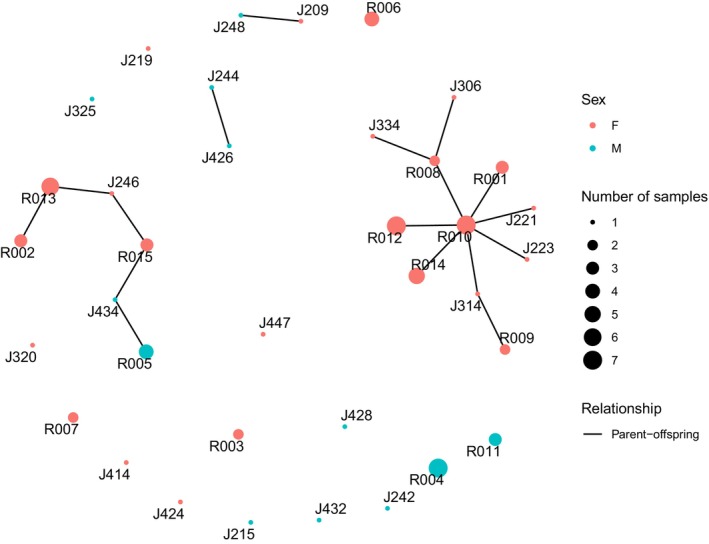
Network of parent‐offspring relationships among biopsy samples collected from the Joint Base Elmendorf‐Richardson (JBER). Each individual black bear is represented by a point (node) labeled with the unique bear identifier, whose sex was determined using the *SRY* locus. The size of each node represents the number of times an individual was identified in the dataset (i.e., the number of samples with matching microhaplotype genotypes). Parent‐offspring pairs are connected by lines.

Patterns of relatedness were very different for biopsy samples collected from JBER. Out of 82 successfully sequenced samples, we identified 36 individual bears, composed of twice as many females as males (*n*_female_ = 24, *n*_male_ = 12). In addition, 15 of the 36 individuals had genotypes that matched another sample perfectly, indicating that these individuals were sampled multiple times (range = 2–7 times) on JBER. However, there was no statistically significant relationship between the sex of an individual and the number of times it was sampled (*Χ* ^2^ = 2.06, df = 1, *p* = 0.15). Finally, we identified 42 pairs of possible first‐order relatives in the JBER samples (parent‐offspring pairs = 17, full‐sibling pairs = 25) that were distributed across several family networks (Figure 6 and Figure S10; Table S7).

## 4 Discussion

### 4.1 Performance of GT‐Seq Panel

We present here an amplicon sequencing panel for Alaska black bears that can be used to identify individuals and first‐order relatives. Our results demonstrate that the GT‐seq panel had high genotyping success in both tooth and biopsy samples, and that heterozygote genotypes were characterized by even allelic depth ratios (Figure 4B). Furthermore, genotypes were reproducible, as the genotyping discrepancy rate was 0.003% for samples that were sequenced in replicate. By targeting SNPs with high heterozygosity and treating multiple SNPs in a sequence read as multiallelic microhaplotypes, we achieved ample statistical power for both individual identification and kinship inference. For example, the false positive rate for identifying two unrelated pairs of samples as the same individual or as a pair of first‐order relatives was very small; simulations demonstrated that at a false negative rate of 0.01, our GT‐seq panel yielded a false positive rate of 1.4×10^−62^ for identifying pairs of resampled individuals, 1.5×10^−10^ for parent–offspring pairs, and 2.3×10^−7^ for full‐sibling pairs.

As others have discussed (Bravington, Skaug et al. 2016; Baetscher et al. 2018), high statistical power is important in the context of studies that involve large sample sizes and therefore generate a large number of pairwise comparisons, such as genetic mark‐recapture or close‐kin mark‐recapture methods that are used to estimate census population size. For example, a typical study design including 2500 samples (i.e., Moore et al. 2014) would produce 6.25 million pairwise comparisons of individuals. Under these conditions and using the false positive rates estimated for the GT‐seq panel, our simulations suggest that no unrelated individuals would be identified as resampled individuals or parent‐offspring pairs and only two pairs of unrelated individuals would be misidentified as full‐siblings. We anticipate that the robust statistical power of the GT‐seq panel for individual identification will be beneficial when genotyping DNA from scat or hair, as previous studies have shown that missing data (Burgess et al. 2022) and genotyping discrepancy rates (Burgess et al. 2022; Arpin et al. 2024) increase with the DNA degradation that is characteristic of some types of non‐invasive samples.

While the probability of misidentifying unrelated individuals as first‐order relatives was low, there was some overlap in the log‐likelihood ratio distributions of simulated full‐siblings and half‐siblings (Figure 5B,C, Figure S8). Previous studies have suggested that reliable identification of half‐sibling pairs likely requires thousands of markers and knowledge of the position of the markers along chromosomes in order to account for the coinheritance of markers due to physical linkage (Bravington, Skaug et al. 2016). Our study design allowed us to explore the effect of the marker number on kinship inference as the same set of hunter‐harvested samples was analyzed separately using 170 GT‐seq microhaplotypes or 6630 RAD‐seq SNPs. Concordance between analyses was very high (Figure 4A,B). However, there was evidence of a possible false positive full‐sibling pair in the GT‐seq data set, as those individuals were identified as likely second‐order relatives using RAD‐seq loci.

One drawback of our approach is that the ascertainment samples used to identify loci for the GT‐seq panel were collected from the northern edge of the species distribution. As black bears exhibit significant population structure across North America (Puckett et al. 2015), it is possible that the GT‐seq panel would be impacted by ascertainment bias if it is used to estimate kinship in populations outside of Alaska. In other words, the SNPs we identified in this study may not be sufficiently polymorphic to identify first‐order relatives in highly diverged populations from the continental United States or Mexico. Future research that evaluates the heterozygosity of the GT‐seq panel across the species range would be beneficial for assessing the limits of the panel's broader use, similar to what has been done for other microsatellites (Paetkau and Strobeck 1998) and SNP panels (Kopatz et al. 2024) used in wildlife management.

### 4.2 JBER Case Study

As a proof of concept, we used the GT‐seq panel to estimate the number of individuals and first‐order relatives from biopsy samples collected on JBER in Anchorage. We found that 44% of samples came from 36 distinct individuals, while the remainder originated from bears that were sampled multiple times. These results were similar to Muhlenbruch (2022), who identified 45 individual bears on the same military base over a two‐year period using microsatellites. Both studies sampled individuals opportunistically and thus included individuals that were sampled in natural habitats while foraging on wild foods as well as bears that were encountered at urban sites while feeding on anthropogenic food sources such as garbage. Flexibility in resource use is a well‐known characteristic of black bears (Baruch‐Mordo et al. 2014), and individual tracking of bears by the Alaska Department of Fish and Game has demonstrated that individuals move between wild and urban environments on JBER (Saalfeld and Farley 2015). Taken together, our results indicate that numerous black bears access both wild habitats and anthropogenic food resources available on the military base. This has implications for the management of bear attractants and provides baseline data for mitigating human‐bear conflicts.

In contrast to the nearly even sex ratio and small number of close relatives encountered in the hunter‐harvested samples, we detected many first‐order relatives and approximately twice as many females as males on JBER. While there was no statistically significant relationship between the sex of an individual and the number of times it was sampled, this result was likely due to the small sample size of our study. Our results indicated that a subset of individuals from JBER had connections to numerous first‐order relatives. For example, a single female (R010, Figure 6) was sampled multiple times throughout the study and was identified in ~40% of parent‐offspring pairs. Unfortunately, we do not have information on the relative ages of individuals in our study, so we cannot identify with confidence which individuals are parents and which are offspring. However, we infer that this network of related bears likely represents a female (R010) with multiple female cubs, as subsets of individuals in the parent‐offspring network were also identified as full‐sibling pairs (Figure S10). Past studies have shown that adult females tolerate female offspring in their home range (Rogers 1987). Furthermore, both genetic relatedness (Moyer et al. 2006) and food availability (Moyer et al. 2007) influence the spatial organization of home ranges, and it has been shown that areas with high food production are used by multiple bears with overlapping home‐range core areas (Powell 1987; Samson and Huot 2001). Our findings suggest that the military base has food resources to support multiple generations of related individuals, which otherwise might disperse farther if anthropogenic foods were unavailable.

### 4.3 Black Bear Population Structure Using RAD‐Seq

Our analysis of black bear population structure using genomic RAD‐seq data provided evidence of three population groups in Southcentral Alaska that are distributed in different geographic regions: Anchorage, the Kenai Peninsula, and Prince William Sound. This spatial differentiation is consistent with a previous microsatellite study (Robinson et al. 2007), suggesting that overall patterns of population structure have remained stable over the last decade. Using different marker sets and analytical methods, both studies observed a genetic discontinuity between eastern Prince William Sound and Kenai Peninsula black bear populations. This geographic region includes multiple geographic features that are likely barriers to dispersal, such as glaciated mountains, large fjords, and strong tidal currents. In other parts of the species range, such as the American Southwest and Mexico, rugged terrain has been shown to restrict gene flow between black bear populations (Gould et al. 2022). Given the limited geographic extent of sample collections in our study and in Robinson et al. (2007), it is not clear whether population structure in Southcentral Alaska is primarily driven by contemporary barriers to gene flow or reflects earlier phylogeographic differentiation. This uncertainty arises, in part, from the fact that black bear population structure in Alaska is poorly understood. To date, the most geographically comprehensive study of black bear population structure and phylogeography in North America (Puckett et al. 2015) did not include individuals collected north of the Kenai Peninsula. To identify the mechanisms shaping the genetic structure of Alaska black bears, future investigations could implement a landscape genomics approach to contextualize findings across larger spatial scales, including mainland Alaska.

While pairwise *F*_ST_ values demonstrated significant allele frequency differences between population groups, the presence of admixed individuals in all groups indicated that gene flow occurred in previous generations. Admixture was most prevalent between the Anchorage and Kenai Peninsula populations, indicating increased connectivity between these groups relative to the Prince William Sound population, a result which is in agreement with previous work (Robinson et al. 2007). The high‐resolution genomic data also enabled us to identify bears that dispersed from their natal population to a genetically distinct population group. For example, one male and one female bear, with ancestry predominantly from the Anchorage population, were harvested on the Kenai Peninsula. Additionally, we identified one male bear with Prince William Sound ancestry that was harvested on the eastern Kenai Peninsula. These findings are consistent with previous observations of frequent long‐distance dispersal by male bears (Schwartz and Franzmann 1992; Moore et al. 2014) and occasional long‐distance dispersal by female bears (Moore et al. 2014) from their natal ranges.

### 4.4 Future Applications

Southcentral Alaska contains many wild habitats and wildlife species, but the region is also home to a majority of the state's human population. As human encroachment into undeveloped land continues, the monitoring of bear populations is a priority for wildlife managers (Stantorf and Spivey 2022). However, factors such as foliage density, canopy density, and air space classification limit the effectiveness of traditional aerial population estimation techniques (Stantorf and Spivey 2022). Consequently, the management of black bears within Southcentral Alaska is currently informed through the monitoring of hunter harvest and other mortality events (e.g., roadkill, agency kills, and defense of life or property), with a goal of maintaining the adult female harvest below a specific threshold (Herreman 2022; Stantorf and Spivey 2022; Westing 2022).

We anticipate that our GT‐seq panel will be useful to future investigations of Alaska black bears. For example, the panel's high statistical power for individual identification and parentage analysis will be beneficial if genetic mark‐recapture analyses are used to estimate population sizes and demographic parameters. This method could also be applied to quantifying individual dispersal and foraging patterns or testing hypotheses about social learning within family groups. Finally, the accurate identification and enumeration of bears involved in human‐wildlife conflicts can assist managers as they design and assess mitigation strategies and communicate to stakeholders.

## Author Contributions

**Eleni L. Petrou:** conceptualization (equal), data curation (equal), formal analysis (lead), investigation (lead), methodology (lead), project administration (equal), validation (lead), visualization (lead), writing – original draft (lead). **Colette D. Brandt:** conceptualization (lead), data curation (equal), funding acquisition (lead), investigation (equal), project administration (equal), writing – review and editing (equal). **Timothy J. Spivey:** conceptualization (lead), data curation (equal), formal analysis (supporting), funding acquisition (supporting), investigation (lead), project administration (supporting), writing – original draft (supporting), writing – review and editing (equal). **Kristen M. Gruenthal:** formal analysis (supporting), investigation (supporting), methodology (supporting), writing – original draft (supporting), writing – review and editing (equal). **Cherie M. McKeeman:** formal analysis (supporting), methodology (supporting), visualization (supporting), writing – review and editing (equal). **Sean D. Farley:** conceptualization (equal), data curation (supporting), investigation (supporting), writing – review and editing (equal). **David Battle:** data curation (supporting), investigation (supporting), writing – review and editing (equal). **Cory Stantorf:** data curation (supporting), investigation (supporting), writing – review and editing (equal). **Andrew M. Ramey:** conceptualization (lead), funding acquisition (equal), methodology (supporting), project administration (lead), supervision (lead), writing – review and editing (equal).

## Conflicts of Interest

The authors declare no conflicts of interest.

## Supporting information


Figure S1.
Figure S2.
Figure S3.
Figure S4.
Figure S5.
Figure S6.
Figure S7.
Figure S8.
Figure S9.
Figure S10.

Table S1.
Table S2.
Table S3.
Table S4.
Table S5.
Table S6.
Table S7.

## Data Availability

Restriction site‐associated sequencing data reported in this study have been deposited in the National Center for Biotechnology Information Sequence Read Archive (https://www.ncbi.nlm.nih.gov/sra) and are accessible through BioProject ID PRJNA1199053. Sample metadata are available in the Tables S1–S7, and genotyping data from this study are publicly available in Petrou et al. (2025).
