# Supplementary material for: Development of a Genotyping‐in‐Thousands by Sequencing (GT‐Seq) Panel for Identifying Individuals and Estimating Relatedness Among Alaska Black Bears ( Ursus americanus )
Source: Ecol Evol. 2025 Apr 11;15(4):e71273. doi: 10.1002/ece3.71273 (PMC11992360; doi:10.1002/ece3.71273)
Supplement: Supplementary file 1 — Figure S1. Figure S2. Figure S3. Figure S4. Figure S5. Figure S6. Figure S7. Figure S8. Figure S9. Figure S10. [file ECE3-15-e71273-s001.docx]

**Supplemental Information: Figures**


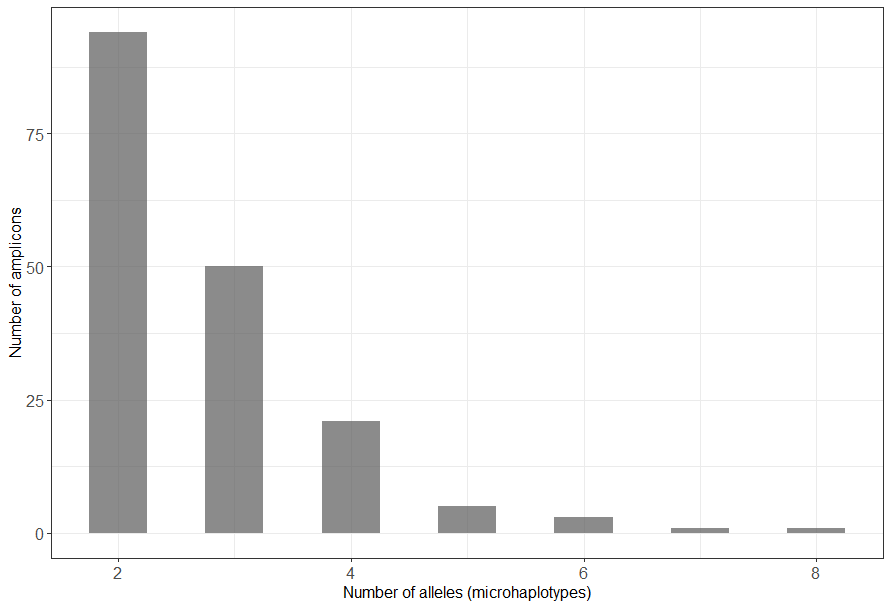
Figure S1. Distribution of the number of microhaplotypes present in 170 loci included in panel, based on hunter-harvested black bear samples genotyped with restriction-site associated DNA sequencing. There were two to eight microhaplotype alleles present per candidate genotyping-in-thousands by sequencing (GT-seq) locus.


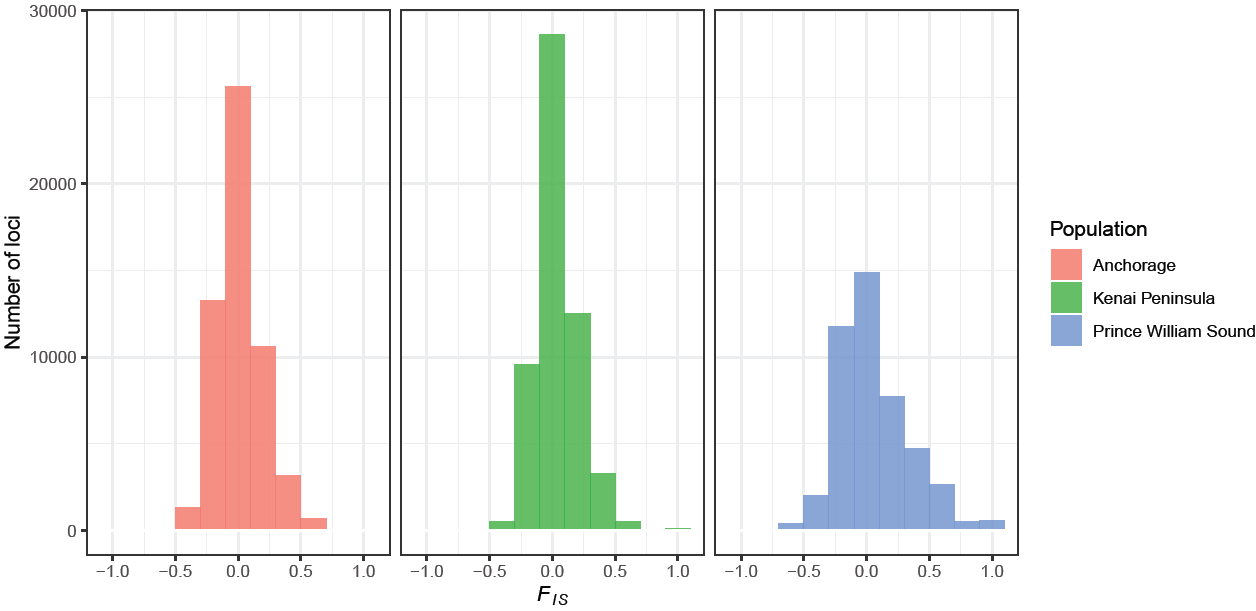
Figure S2. Distribution of per-locus inbreeding coefficient (*F*_IS_) for hunter-harvested black bears genotyped at 56K SNPs using restriction-site associated DNA sequencing. The different panels display results for each population separately.


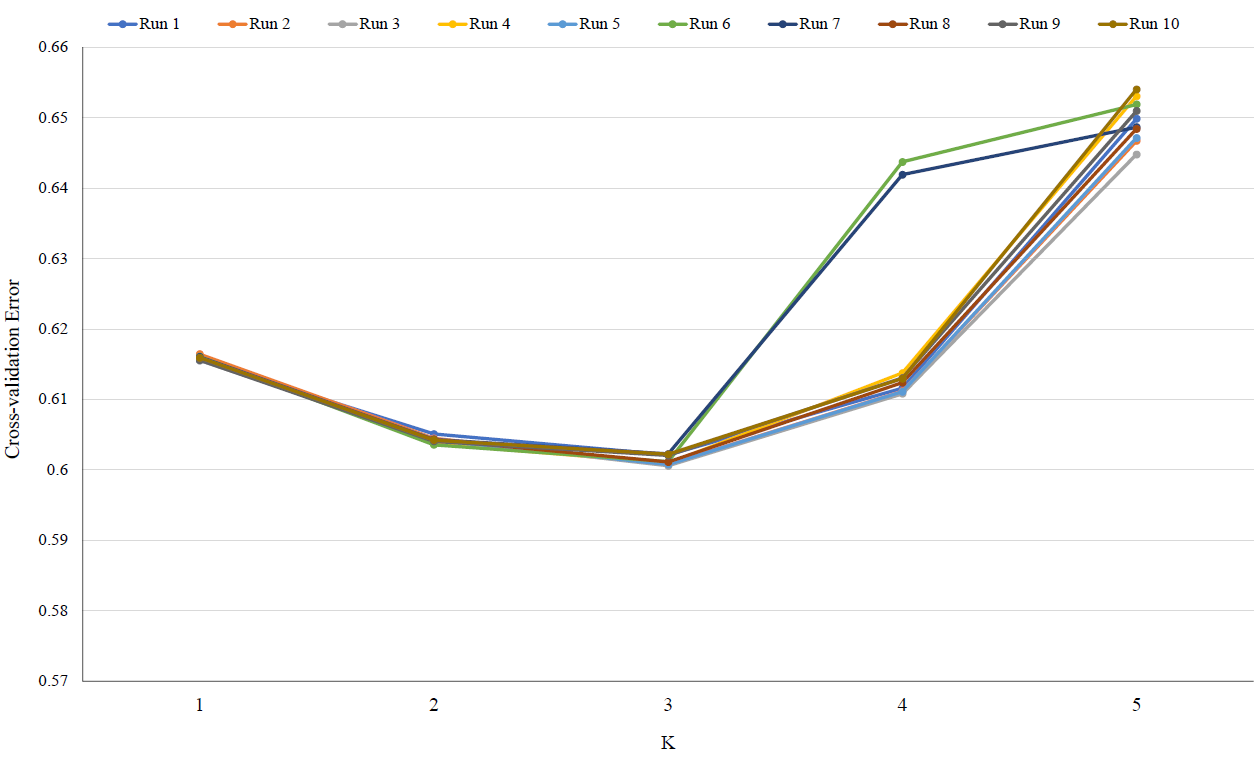


Figure S3. Cross-validation errors from admixture analysis for 6,831 SNPs genotyped in 85 individuals using RAD sequencing. For this analysis, SNPs were pruned for linkage disequilibrium, a random seed was generated from the time of the run, and the program was run 10 times for values of *K* from 1 to 5.


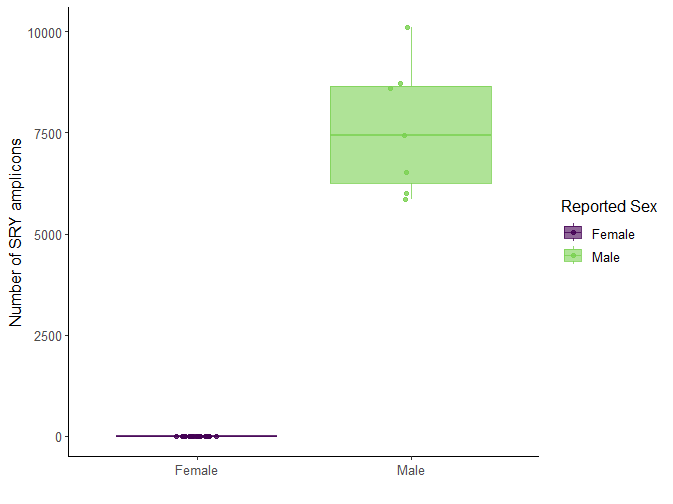


Figure S4. The number of *SRY* gene amplicons in DNA extracts from black bears whose sex was previously identified (“Reported Sex”) using an independent sex-linked marker (*zfx/y*).


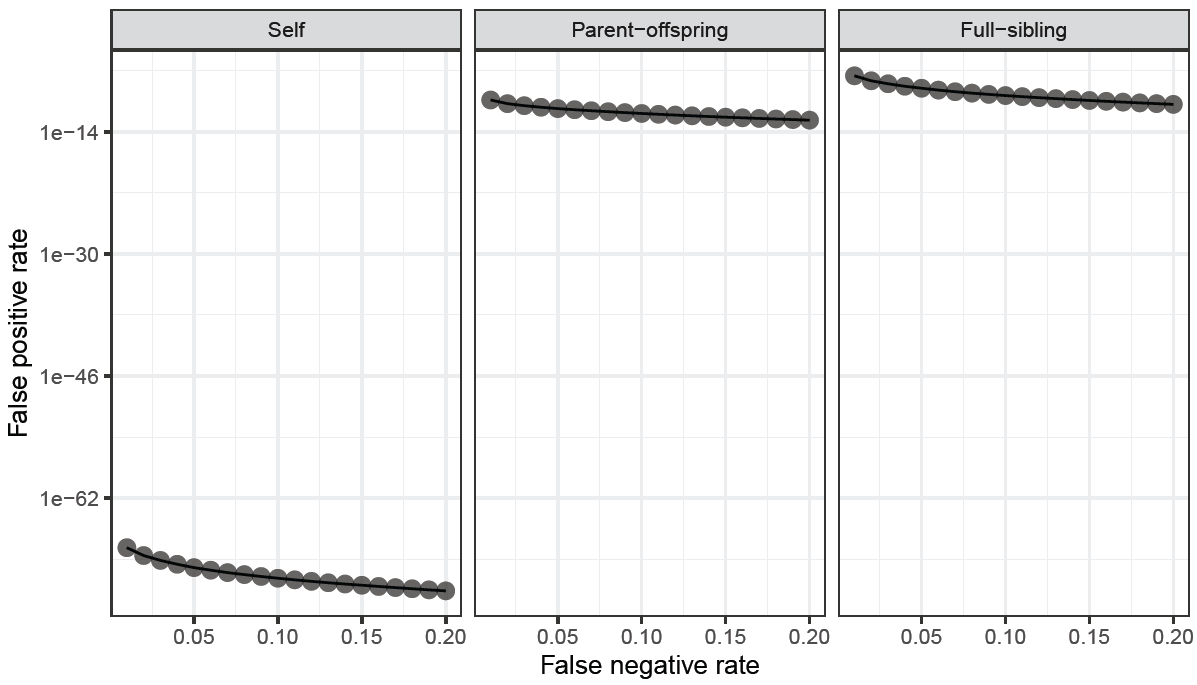
Figure S5. Simulated false-positive rates (FPR) as a function of the false negative rate using microhaplotype allele frequencies of hunter-harvested black bears genotyped with GT-seq. Each panel shows the FPR for a identifying an unrelated pair of individuals as monozygotic twins (or self), parent-offspring, or full-sibling pairs.


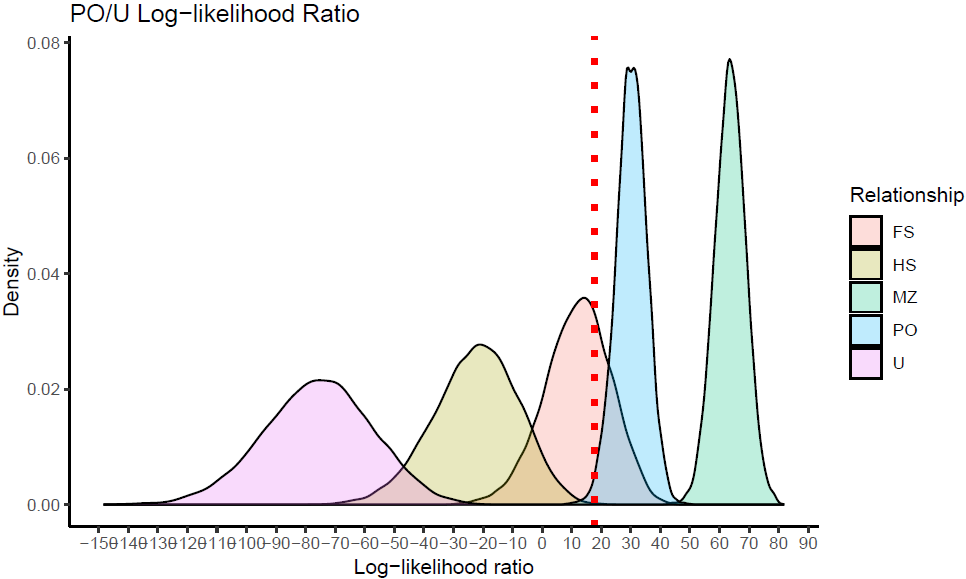


Figure S6. Distribution of the PO/U log-likelihood ratio for simulated pairs of monozygotic twins (MZ), parent-offspring (PO), full-siblings (FS), half-siblings (HS), and unrelated individuals (U). Simulations were based on the allele frequencies of hunter-harvested black bears genotyped at 170 microhaplotype loci. The dotted red line indicates the log-likelihood ratio threshold for distinguishing PO pairs from U pairs, assuming a false negative rate = 0.01.


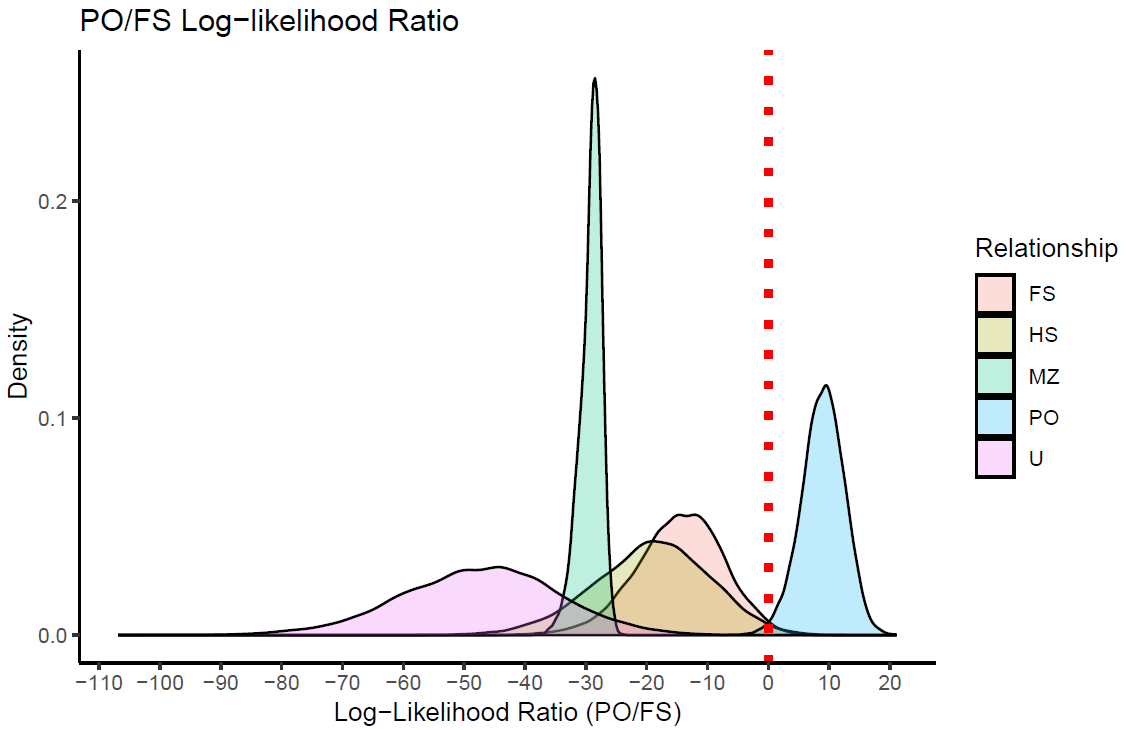


Figure S7. Distribution of the PO/FS log-likelihood ratio for simulated pairs of monozygotic twins (MZ), parent-offspring (PO), full-siblings (FS), half-siblings (HS), and unrelated individuals (U). Simulations were based on the allele frequencies of hunter-harvested black bears genotyped at 170 microhaplotype loci. The dotted red line indicates the log-likelihood ratio threshold for distinguishing PO pairs from FS pairs, assuming a false negative rate = 0.01.


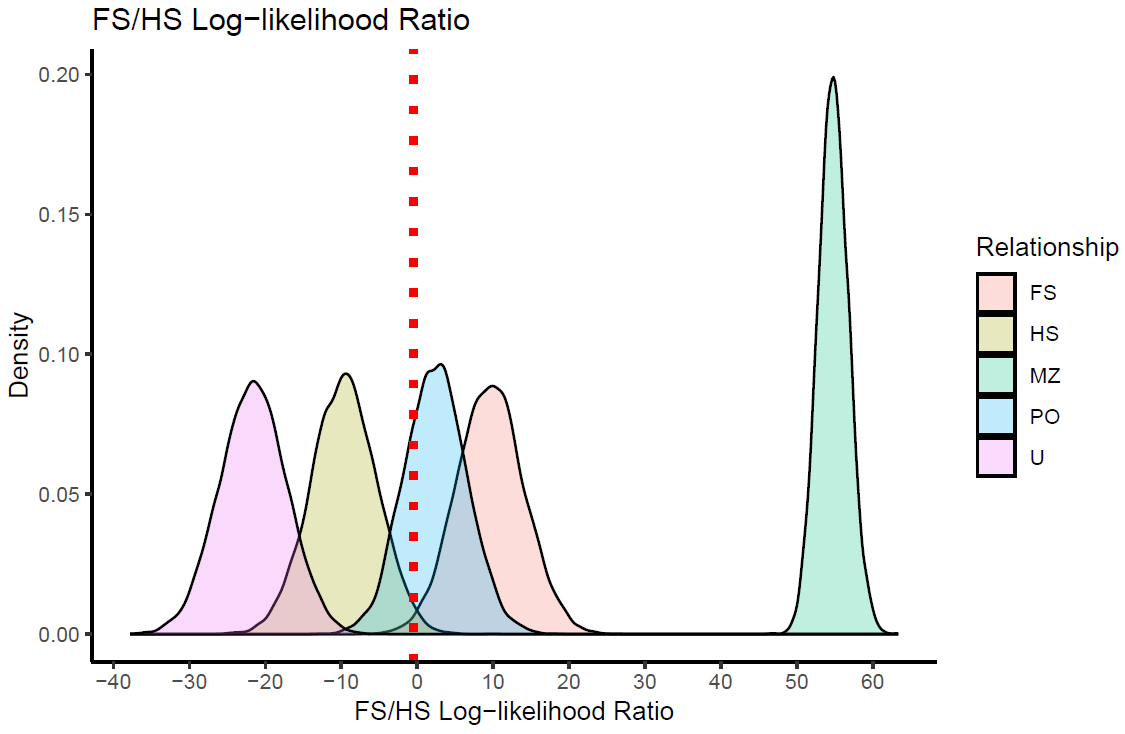


Figure S8. Distribution of the FS/HS log-likelihood ratio for simulated pairs of monozygotic twins (MZ), parent-offspring (PO), full-siblings (FS), half-siblings (HS), and unrelated individuals (U). Simulations were based on the allele frequencies of hunter-harvested black bears genotyped at 170 microhaplotype loci. The dotted red line indicates the log-likelihood ratio threshold for distinguishing FS pairs from HS pairs, assuming a false negative rate = 0.01.


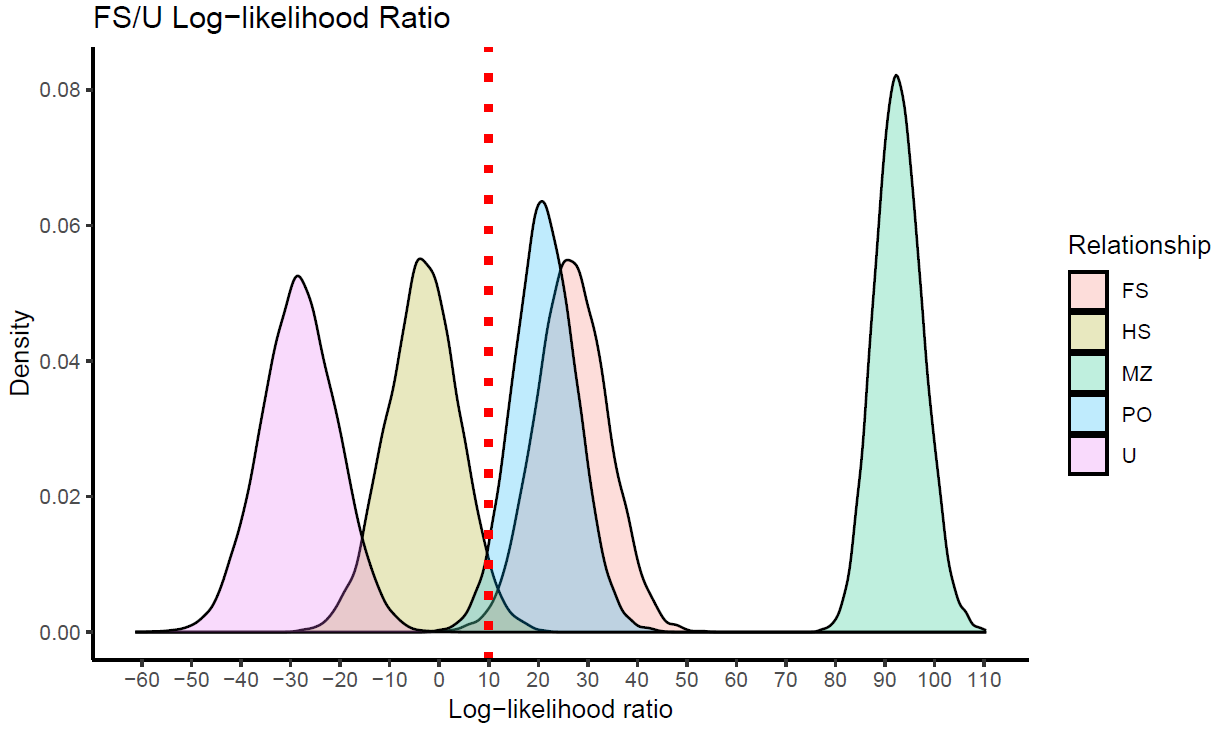


Figure S9. Distribution of the FS/U log-likelihood ratio for simulated pairs of monozygotic twins (MZ), parent-offspring (PO), full-siblings (FS), half-siblings (HS), and unrelated individuals (U). Simulations were based on the allele frequencies of hunter-harvested black bears genotyped at 170 microhaplotype loci. The dotted red line indicates the log-likelihood ratio threshold for distinguishing FS pairs from U pairs, assuming a false negative rate = 0.01.


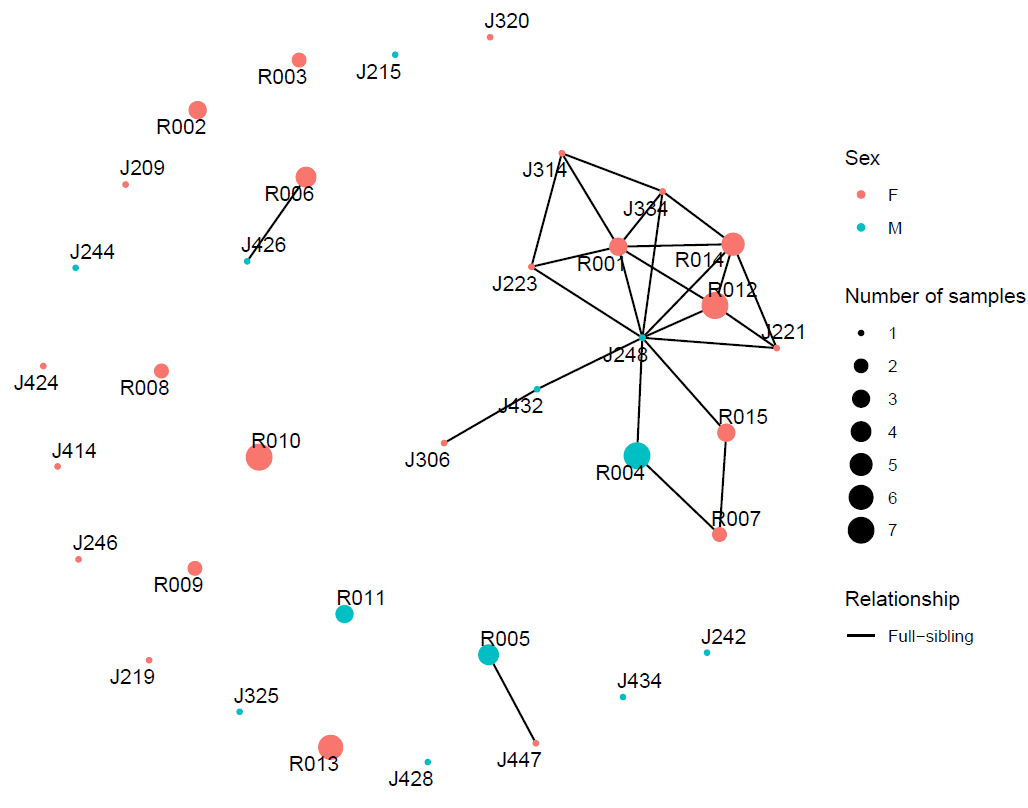


Figure S10. Network of full-sibling relationships among black bear biopsy samples collected from the Joint Base Elmendorf-Richardson . Each individual bear is represented by a point (node) labelled with the unique bear identifier, whose sex was determined using the SRY locus. The size of each node represents the number of times an individual was identified in the dataset (i.e., the number of samples with matching microhaplotype genotypes). Full-sibling pairs are connected by lines.
